# A Simple Sandwich Electrochemical Immunosensor for Rapid Detection of the Alzheimer’s Disease Biomarker Tau Protein

**DOI:** 10.3390/bios14060279

**Published:** 2024-05-29

**Authors:** Mingzhu Yang, Yihong Chen, Hongyu Sun, Dujuan Li, Yanbin Li

**Affiliations:** 1 Ministry of Education Engineering Research Center of Smart Microsensors and Microsystems, College of Electronics and Information, Hangzhou Dianzi University, Hangzhou 310018, China; 231040025@hdu.edu.cn (M.Y.); shy19816896756@163.com (H.S.); 2 Zhejiang College of Construction, Hangzhou 311231, China; chenyihong@zjjs.edu.cn; 3 School of Automation, Hangzhou Dianzi University, Hangzhou 310018, China; 4 Department of Biological and Agricultural Engineering, University of Arkansas, Fayetteville, AR 72701, USA; yanbinli@uark.edu

**Keywords:** tau protein, Alzheimer’s disease (AD), sandwich, immunosensor, impedance

## Abstract

As a typical biomarker of Alzheimer’s disease, rapid and specific detection of tau protein can help improve the early diagnosis and prognosis of the disease. In this study, a simple sandwich electrochemical immunosensor was developed for rapid detection of tau protein. Primary monoclonal antibodies (mAb1) against the middle domain of tau protein (amino acids 189–195) were immobilized on the gold electrode surface through a self-assembled monolayer (SAM) of 3,3′-dithiobis (sulfosuccinimidyl propionate) (DTSSP). Then the tau protein was captured through the specific adsorption between the antigen and the antibody, resulting in a change in the impedance. Secondary monoclonal antibodies (mAb2) against the N-terminal region of tau protein were used for further amplification of the binding reaction between mAb1 and tau protein. A linear correlation between the total change in impedance and the logarithm of tau concentration was found from 2 × 10^−6^ mg mL^−1^ to 2 × 10^−3^ mg mL^−1^, with a detection limit as low as 1 × 10^−6^ mg mL^−1^. No significant interference was observed from human serum albumin. Furthermore, the fabricated sandwich immunosensor successfully detected target tau protein in artificial cerebrospinal fluid (aCSF) samples, indicating good potential for clinical applications in the future.

## 1. Introduction

Biomarkers in body fluids, such as cerebrospinal fluid (CSF) and plasma, serve as indicators of physiological or pathological processes and have become an indispensable part of the clinical diagnosis and prognosis of neurodegenerative diseases (NDDs). Research also suggests that salivary biomarkers can be used for screening and diagnosing NDDs [1]. Tau protein is a biomarker for Alzheimer’s disease and is abundant in brain neurons and bound to neuronal microtubule [2,3,4], which could maintain neuron growth, neuron polarity, and intracellular material transport. In healthy conditions, tau protein stably existed in neuronal cells, but when tau underwent complex post-translational modifications (PTMs) that caused its dissociation from microtubules and aggregation into neurofibrillary tangles and paired helical filaments [5,6], neurological diseases such as AD were caused [7].

Nowadays, about fifty million people worldwide suffer from neurodegenerative diseases, with more than ten million cases discovered annually, of which nearly 70% are people with AD [8,9]. AD is a degenerative disease with the characteristics of memory loss and neuronal cell death [10,11]. In the early stages, there is a problem in patients with memory and language that gradually develops to the point where they cannot take care of themselves mentally and physically [12]. Hence, there is an urgent need to find ways to provide clues towards an early and precise diagnosis. Human tau protein is one of the most advanced and accepted biomarkers for AD and tauopathies in general [13,14,15].

The most frequently used strategy is the direct detection of tau protein by sandwich enzyme-linked immunosorbent assay (ELISA); however, the applicability of ELISA tests for point-of-care was limited as they usually required large and expensive instrumentation and additional reagents, were time-consuming with time-to-results of several hours, and were limited in sensitivity [16,17]. At the same time, due to their high sensitivity, simple use, rapid response, and low cost, biosensors have been extensively applied in clinical diagnosis and biomedical studies [18,19,20,21,22]. Typically, tau protein is detected by immunosensing methods [23]. In order to improve the detection signal, sandwich detection strategies and nanomaterials are widely incorporated into immune sensors [24,25]. For example, Li Chen et al. [23] introduced the CuInS_2_/ZnS–DA QDs conjugates, which were prepared and used as an efficient redox-mediated fluorescence immunoassay with the use of TYR for the detection of Alzheimer’s disease biomarkers with a detection limit of 9.3 pM. S. Lisi et al. [24] reported that Surface Plasmon Resonance (SPR) coupled to Multi-Walled Carbon Nanotubes (MWCNTs) could enhance the analytical performances of the biosensor by applying a sandwich-like detection strategy, which could exploit tau protein detection at the picomolar level in aCSF. Xu Hun et al. [26] constructed an electrochemical biosensor that used commercial aptamers, antibodies, and protein G labeled with alkaline phosphatase (protein G/AP) coupled with AuNPs/MoSe_2_NSs modified electrodes for the detection of Tau-381 protein. A. Ben Hassine et al. [27] developed an innovative biosensor using a carbon screen-printed electrode (C-SPE) decorated with graphene oxide/Prussian Blue nanocubes (GO/PBNCs) for the selective and sensitive determination of Tau-441 protein. C. A. Razzino et al. [25] prepared a disposable amperometric immunosensor for the sensitive determination of tau protein by implementing a sandwich immunoassay onto SPCEs grafted with p-ABA, modified with a 3D-Au-PAMAM nanocomposite, and involving an HRP-labeled detector antibody. However, the mentioned above sensor technologies still face challenges such as complex material preparation, long detection times, and relatively low sensitivity.

Herein, we report a simple and fast approach to detecting the tau protein. This study developed an immune sandwich impedance sensor for rapid detection of tau protein, which was first modified with mAb1 on the basis of the DTSSP self-assembled monomolecular layer modified onto the surface of the gold electrode, then the tau protein was captured through the specific adsorption between the antigen and the antibody, and finally mAb2 was added to realize further amplification of the signal. Furthermore, electrochemical tests were performed using electrochemical impedance spectroscopy (EIS) and cyclic voltammetry (CV) with Fe(CN)_6_^3−/4−^(1:1) as the probe [28].

## 2. Materials and Methods

### 2.1. Reagents and Materials

The primary antibody (mAb1, monoclonal, clone 39E10 produced in mice) was from Biolegend (San Diego, CA, USA). Human tau protein (tau441, isoform 2N4R, MW 46 kDa) was purchased from Enzo Life Sciences (Lyon, France). Secondary antibody (mAb2, monoclonal, clone Tau12, produced in mice) was acquired from Merck (Daarmstaad, Germany). Human serum albumin (HSA) was taken from Sigma-Aldrich, Milan, Italy. 3,3′-dithiobis (sulfosuccinimidyl propionate) (DTSSP) was from Sigma-Aldrich (St. Louis, MO, USA), Potassium ferrocyanide (K_4_Fe(CN)_6_), potassium ferricyanide (K_3_Fe(CN)_6_), sodium chloride (NaCl), sodium dihydrogen phosphate (NaH_2_PO_4_), disodium hydrogen phosphate (Na_2_HPO_4_), potassium chloride (KCl), magnesium sulphate (MgSO_4_), sodium hydrogen bicarbonate(NaHCO_3_), calcium chloride(CaCl_2_), and glucose were purchased from Sinopharm Chemical Reagent Co., Ltd. (Shanghai, China). The deionized water was supplied by a Millipore Milli-Q system (18.2 ΜΩ cm).

Phosphate-buffered saline (PBS) consisted of 0.296 g of NaH_2_PO_4_·2H_2_O, 2.901 g of Na_2_HPO_4_·12H_2_O, 9 g of NaCl and 1000 mL of ultrapure water, pH 7.2.

aCSF consisted of 150 mM NaCl, 3 mM KCl, 1.4 mM CaCl_2_, 0.8 mM MgCl_2_, 0.8 mM Na_2_HPO_4_, 0.2 mM NaH_2_PO_4_ with 100 mg L^−1^ HSA, pH 7.3.

### 2.2. Fabrication and the Detecting Principles of Immunosensors

Prior to the construction of the sensor, the gold surface was treated with Pirannha solution (3:1 H_2_SO_4_:H_2_O_2_ *v*/*v* %) for 3 min then the gold surface was polished with 0.3 mm, 0.05 mm aluminum powder respectively and cleaned ultrasonically with ultrapure water for 5 min. After each step was completed, the gold surface was cleaned with deionized water. Subsequently, the sensor was tested by EIS and CV with 1 mM Fe(CN_6_)^3−/4−^(1:1)—PBS solution, ensuring that the cyclic voltammetry peak potential difference ΔEp is less than 75 mv, impedance value is less than 200 Ω.

**Figure 1:**
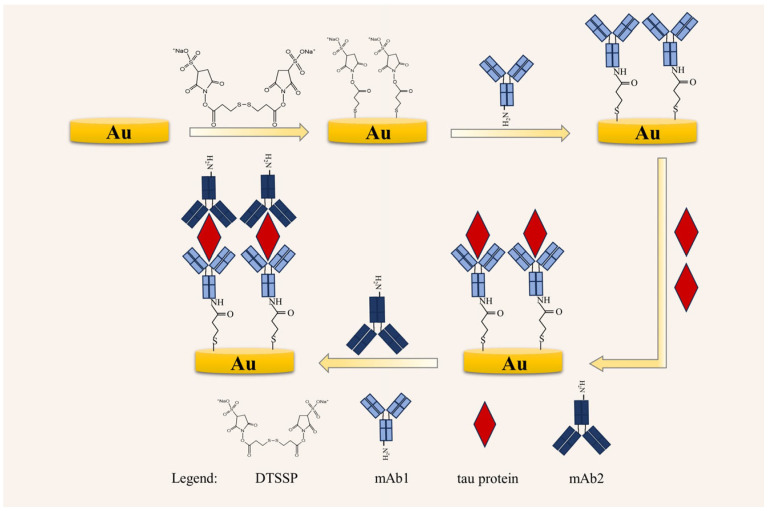
Schematic diagram of sensor fabrication and detection process.

The sensor was fabricated according to the following steps, as shown in Figure 1. Firstly, the polished gold electrode was rinsed with deionized water and then immersed in a 1 mg mL^−1^ DTSSP solution at room temperature for 1 h. The bifunctional molecule DTSSP was modified onto the surface of the gold electrode through the thiol-gold chemical bonds formed between the gold bond and the thiol group. Secondly, the gold electrode surface was rinsed with deionized water and then dried with nitrogen. Then 10 μL of the 0.05 mg mL^−1^ mAb1 was added into the surface to incubate for 1 h at room temperature, to be sure that the gold electrode surface was completely covered by the solution. mAb1 was bound onto the gold surface through alkyl sulfonate-amino groups. After that, the electrode was flushed using 10 mM PBS for 1 min to clear away the unbound antibody. There were unbound sites on the surface of the self-assembled membrane owing to the three-dimensional structure of the protein. To prevent non-specific adsorption, the remaining surface binding sites (not covered by mAb1) were blocked by incubating the sensor in 10 μL of 0.1 M ethanolamine solution (ETA) solution for 1 h at room temperature. Then the electrode was immersed in 10 mM deionized water for 1 min and dried with nitrogen. Thereafter, 10 μL of 0.1 mg mL^−1^ Tau441 protein diluted with PBS was added to the sensor for 1 h. Subsequently, the electrode was rinsed with 10 mM PBS for 1 min and dried with nitrogen. Finally, 10 μL of 0.01 mg mL^−1^ mAb2 was added to the sensor for 1 h, to realize the further amplification of the signal.

### 2.3. Electrochemical Analysis Methods

Electrochemical tests were performed using electrochemical impedance spectroscopy (EIS) and cyclic voltammetry (CV) tests, which were included with the ZAHNER electrochemical workstation. Both EIS and CV measurements were performed in an electrochemical cell, which consisted of a three-electrode system with a 2 mm gold plate electrode for the working electrode, a saturated calomel electrode for the reference electrode, a platinum electrode as the counter electrode, and 20 mL of 1 mM Fe(CN)_6_^3−/4−^ (1:1)-PBS as a redox probe [29]. All the electrochemical tests were performed at room temperature.

EIS measurements were recorded within the frequency range of 0.1 to 100 kHz at open circuit potential and the disturbance voltage at 10 mV. The Nyquist plots (Z′′ and Z′, Z′ is the real part of the impedance and Z′′ is the imaginary part of the impedance, namely R_ct,_, where R_ct_ is the electron transfer impedance) were recorded, and the impedance data were fitted to the equivalent circuit, which was composed of solution impedance, C_dl_, double layer capacitance between electrode and electrolyte, R_S_, electron transfer impedance, R_ct_, and Warburg impedance, Z_w_ [30]. What’s more, CV was carried out by cycling the potential between −0.2 and 0.6 V with a scan rate of 100 mV s^−1^. CV was used to determine whether the peak current change trend was consistent with the impedance change of EIS. The different concentrations of tau protein were tested using different immunosensors, and all experimental studies were performed at least three times.

### 2.4. Preparation of Samples

According to the aCSF formula of Uchida et al. [31], the solution ratio was carried out according to the following concentration: Sodium chloride (NaCl) 118 mM, potassium chloride (KCl) 2.5 mM, magnesium sulfate (MgSO_4_) 3 mM, sodium dihydrogen phosphate (NaH_2_PO_4_) 1.1 mM, sodium bicarbonate (NaHCO_3_) 26 mM, calcium chloride (CaCl_2_) 1 mM, and glucose 11 mM, mixed and stored in a refrigerator at 4 °C.

## 3. Results and Discussion

### 3.1. Fabrication and Working Principles of the Sensor

**Figure 2:**
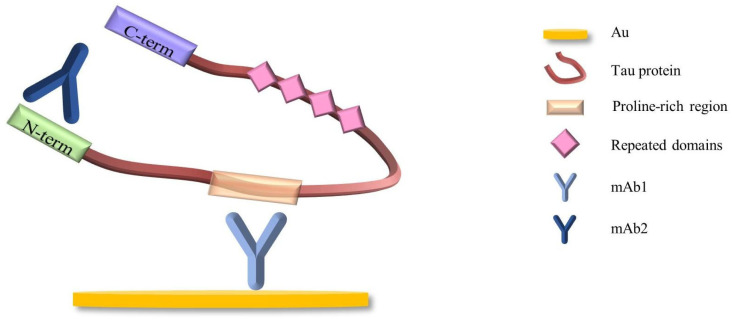
The schematic diagram of the sensor [13].

The schematic diagram of the sensor fabrication is illustrated in Figure 1. First, the bifunctional molecule DTSSP was modified onto the surface of the gold electrode through the thiol-gold chemical bonds formed between the gold bond and the thiol group to form a self-assembled monolayer of DTSSP. Next, mAb1 was modified onto the gold surface through the amide bond formed between the NHS-ester group at the other terminal of DTSSP and the free amino group of mAb1.Due to the three-dimensional structure of protein, there are unbound sites on the surface of the DTSSP self-assembled monolayer. In order to reduce the nonspecific adsorption, ETA was added to block all unreacted active ester groups on the sensor surface. Then, tau protein was captured by specific adsorption between tau protein and mAb1 (Figure 2).

Finally, the specific binding of mAb2 to tau protein further amplified the signal. So far, an immune sandwich impedance sensor has been fabricated. As shown in Figure 2, the sandwich structure consisted of mAb1, tau protein, and mAb2, in which the primary mAb1 recognized the middle domain of tau protein (amino acids 189–195) and was used as a capturing receptor for its direct detection, and mAb2 recognized the N-terminal region of tau protein. The middle domain of tau protein (amino acids 189–195) is a region common to all isoforms of tau protein, so the immunosensor constructed here is suitable for the detection of all isoforms of tau protein.

### 3.2. Characterization of the Sensor

**Figure 3:**
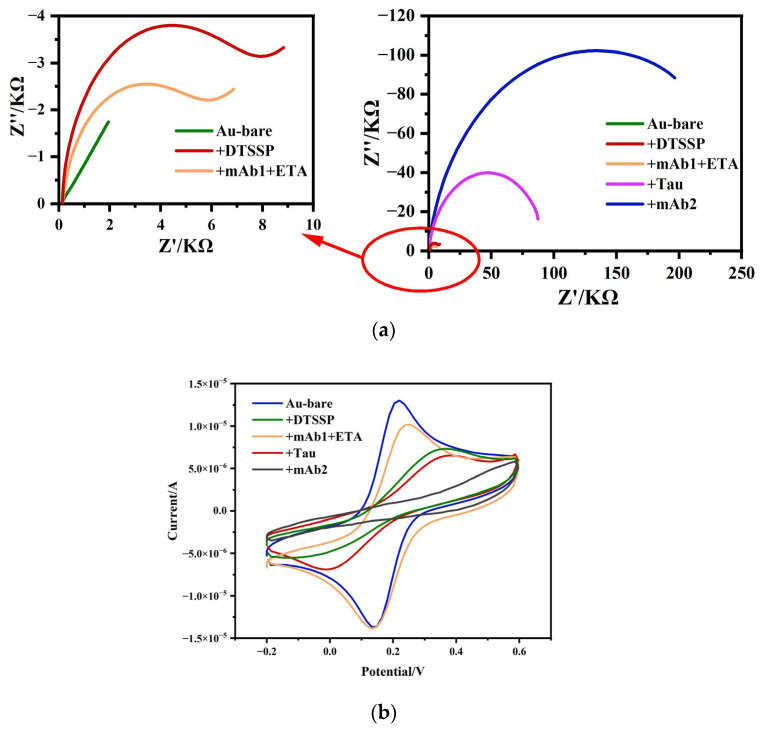
(**a**) Typical Nyquist plots and (**b**) cyclic voltammetry curves of the electrode surface modification, tau protein binding, and secondary antibody binding.

EIS and CV were used to characterize the electron transfer rate change during the stepwise fabrication and detection. As shown in Figure 3, the stepwise fabrication and detection of the immunosensor include DTSSP modification, mAb1 binding and ETA blocking, target Tau protein bonding, and mAb2 adsorption.

As shown in Figure 3a, the surface impedance of the electrode increased after DTSSP was modified onto the surface of the gold electrode. The reason is that the DTSSP self-assembled monolayer on the gold electrode surface reduced the electron transfer rate between the redox probe and the gold electrode surface. The observed decrease in impedance upon addition of mAb1 and ETA can be explained by the positive charge carried by mAb1 itself, which could accelerate the electron transfer rate between the redox probe and the gold electrode surface. After the tau protein was captured, the impedance was higher than that of mAb1 and the ETA procedure, confirming that the Tau441 had been specifically adsorbed to mAb1, which made the self-assembled monolayer on the gold electrode surface more compact and reduced the electron transfer rate between the redox probe and the gold electrode surface. Lastly, the impedance response was further increased as expected when the mAb2 was added to the sensor. This is because the specific binding of the secondary antibody and tau protein formed a more compact self-assembled monolayer on the electrode surface, which further enhanced the difficulty of charge transfer to the electrode surface and reduced the electron transfer rate between the redox probe and the gold electrode surface. The test results showed that the sandwich structure can further amplify the signal.

The cyclic voltammetry curves of the electrode surface modification, tau protein binding, and secondary antibody binding are shown in Figure 3b. The stepwise CV response of the sensor preparation and detection was consistent with the results observed by EIS. A significant decrease in the peak current occurred when DTSSP was modified onto the gold electrode surface, whereas a sharp increase in peak current was observed upon the addition of mAb1 and ETA, which was attributed to the enhanced electron transfer rate due to the positive charge carried by mAb1. The capture of tau protein and the specific binding of mAb2 further decreased the peak current because the stable antibody-antigen complex formed an additional barrier for the redox probe to contact the electrode surface. The CV characterization results were consistent with the EIS results, proving that the immune sandwich impedance sensor was successfully fabricated and can be used for tau protein detection.

### 3.3. Optimization of the Experimental Conditions

In order to optimize biosensor performance and allow the biosensor to detect lower physiological levels of tau protein, the key steps in the functionalization of the biosensor were optimized, including the DTSSP incubation times and the concentration of mAb1, ETA, and mAb2.

#### 3.3.1. Incubation Time of DTSSP

The optimal incubation time of DTSSP was determined by two experimental results, namely, the changes in impedance value caused by different DTSSP incubation time and the impedance response of the final captured tau protein using different DTSSP incubation times.

**Figure 4:**
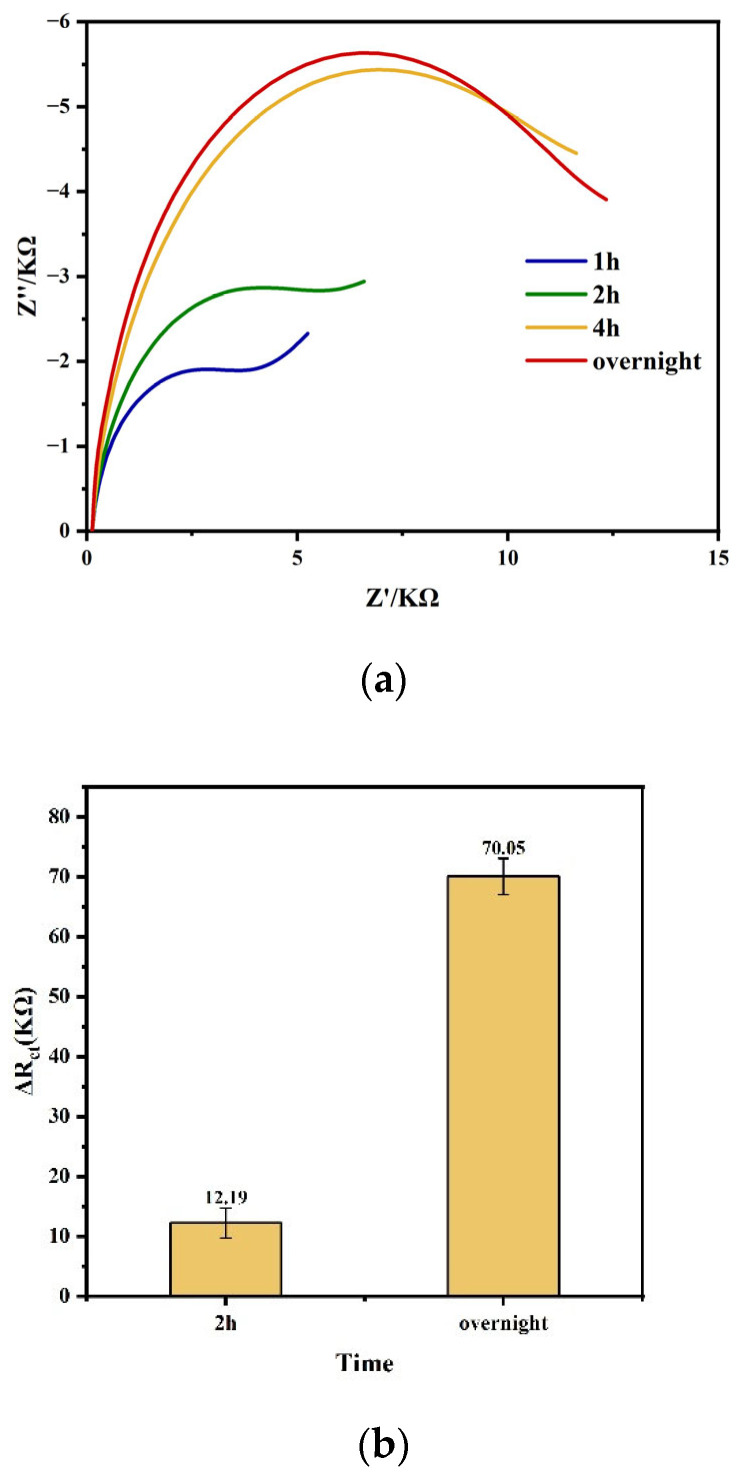
(**a**) Typical Nyquist plots. (**b**) The impedance value of tau protein at different DTSSP incubation time of 2 h and overnight.

Firstly, 1 h, 2 h, 4 h, and overnight were chosen as the incubation times of DTSSP. The typical Nyquist plots and the impedance values are presented in Figure 4a. DTSSP was modified onto the surface of the gold electrode through the thiol-gold chemical bonds, forming a self-assembled monolayer of DTSSP on the gold electrode surface, resulting in a reduction in the rate of electron transfer on the gold electrode surface. As shown in Figure 4, as the modification time increased, the impedance gradually increased. Moreover, it can be seen that when the incubation time reached 4 h, the impedance change closed to the peak value, and increasing the incubation time could not significantly increase the impedance value. The experimental data showed that 4 h was enough for DTSSP to be well modified on the gold electrode surface. However, overnight was a better option considering the experimental timing during actual sensor preparation.

Secondly, different DTSSP incubation times of 2 h and overnight were chosen to fabricate the sensor and capture the protein, respectively. The experiment compared the impedance response of two sensors to tau protein prepared with different incubation times. As shown in Figure 4b, we can see that when the DTSSP incubation time was changed from 2 h to overnight, the shift in impedance was quite large after the tau441 protein capture. Therefore, overnight was selected as the optimal incubation time for DTSSP in sensor fabrication.

#### 3.3.2. Optimization of the mAb1 Concentration

The mAb1 concentration was optimized through comparative experiments. The experiment included a comparison of the impedance signals of mAb1 itself at different concentrations and a comparison of its impedance response values for capturing tau protein.

**Figure 5:**
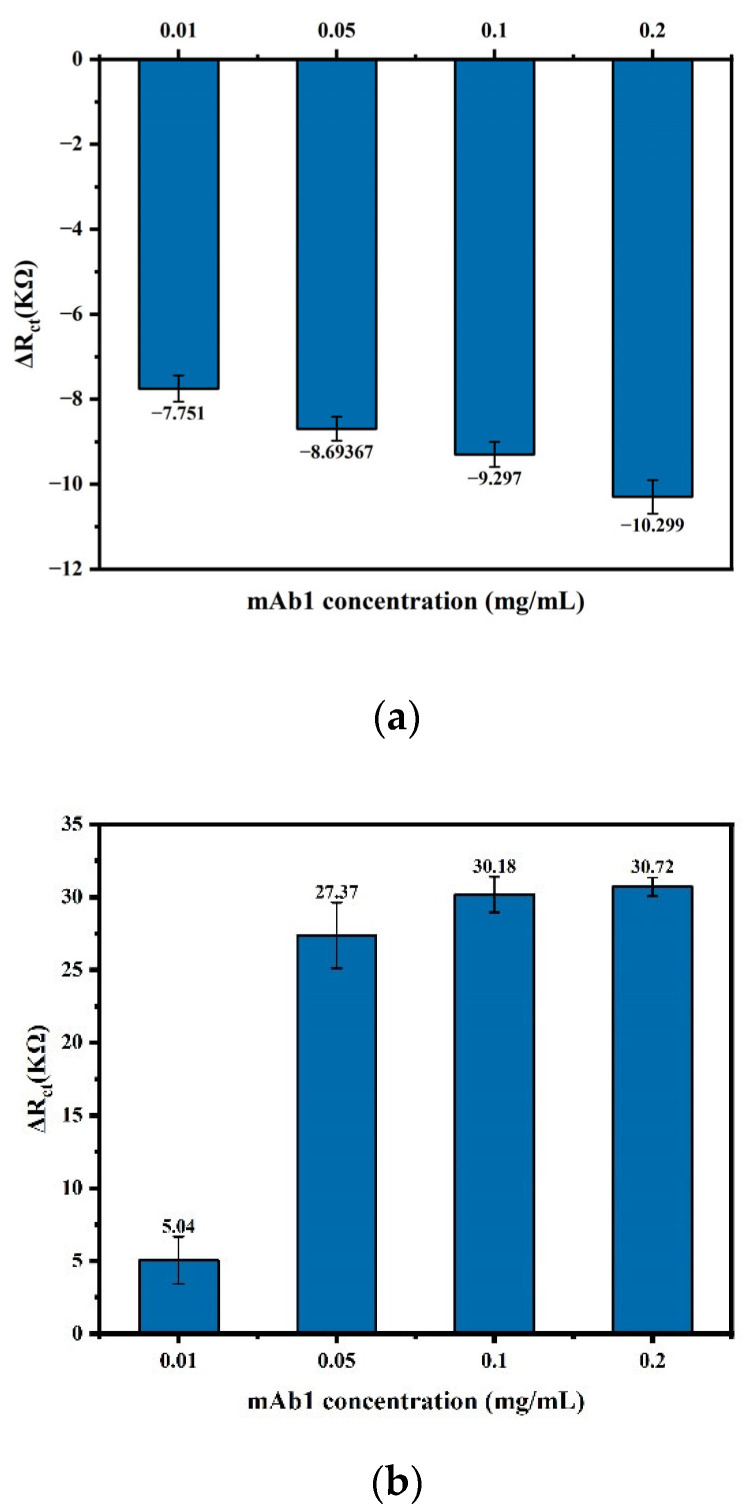
(**a**) The impedance values before and after antibody binding. (**b**) The impedance change caused by the mAb1 capturing the same concentration of tau protein.

Firstly, different concentrations of mAb1 (0.01, 0.05, 0.1, 0.2 mg mL^−1^) were selected and added to the sensor, and the impedance responses caused by them were recorded. The impedance changes before and after mAb1 binding are shown in Figure 5a. The results showed that as the concentrations of mAb1 and ETA increased, the impedance value of the modified sensor gradually increased. When the mAb1 concentration was 0.2 mg·mL^−1^, the impedance value increased the most.

The cost of the sensor is one of the important factors affecting the practical application of the sensor. Therefore, it is also an important consideration in sensor preparation. In the preparation of the immunosensor in this study, the price of the antibody accounts for a large proportion of the sensor cost. Therefore, we further tested and compared the impedance changes caused by different concentrations of mAb1 capturing the same concentration of tau protein (i.e., the target signal after the sensor preparation was completed). The results are shown in Figure 5b.

From the experimental data in Figure 5a,b, it is found that when the antibody concentration is doubled (for example, from 0.05 to 0.1, and from 0.1 to 0.2), the signal of capturing tau protein does not increase proportionally. Moreover, when the antibody concentrations were 0.05, 0.1, and 0.2, respectively, the target response signals of the corresponding sensors were almost at the same level. As can be seen from the schematic diagram of the sensor structure in Figure 2, when the mAb1 antibody concentration is very high and the sensor surface is very densely modified, the three-dimensional structure of the protein prevents it from binding to all antibodies. This results in some mAb1 antibodies being left free. Therefore, considering the response signal of the sensor and the preparation cost, 0.05 mg·mL^−1^ was selected as the optimal concentration of the primary antibody, mAb1.

#### 3.3.3. Optimization of ETA Concentration

**Figure 6:**
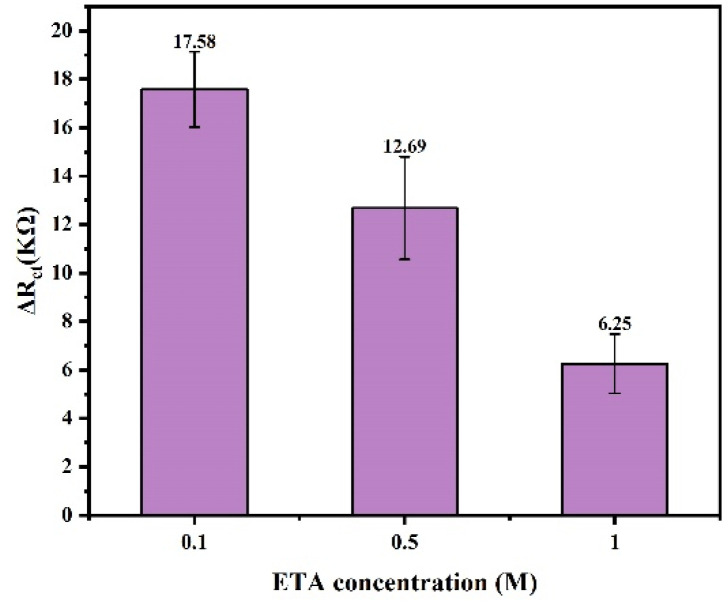
The impedance values after adding different concentrations of ETA solutions.

To prevent non-specific adsorption, the remaining surface binding sites of DTSSP (not covered by mAb1) were blocked by incubating the sensor in ETA. Therefore, in this study, the concentration of ETA was also optimized to prevent the non-specific signals caused by the binding of tau protein to DTSSP. ETA at different concentrations (0.1, 0.5, 1 M) were added to the sensor. The impedance values before and after adding ETA are shown in Figure 6.

The test results showed that as the concentration of ETA increases, the impedance value decreases, which proved that most sites on DTSSP were blocked by ETA, and the blocking effect of 1 M concentration was better, so the best choice of ETA concentration was 1 M.

#### 3.3.4. Optimization of mAb2 Concentration

As a secondary antibody, mAb2 can specifically bind to the captured tau protein and specifically amplify the target signal. A comparative experimental study was conducted on the concentration of mAb2.

**Figure 7:**
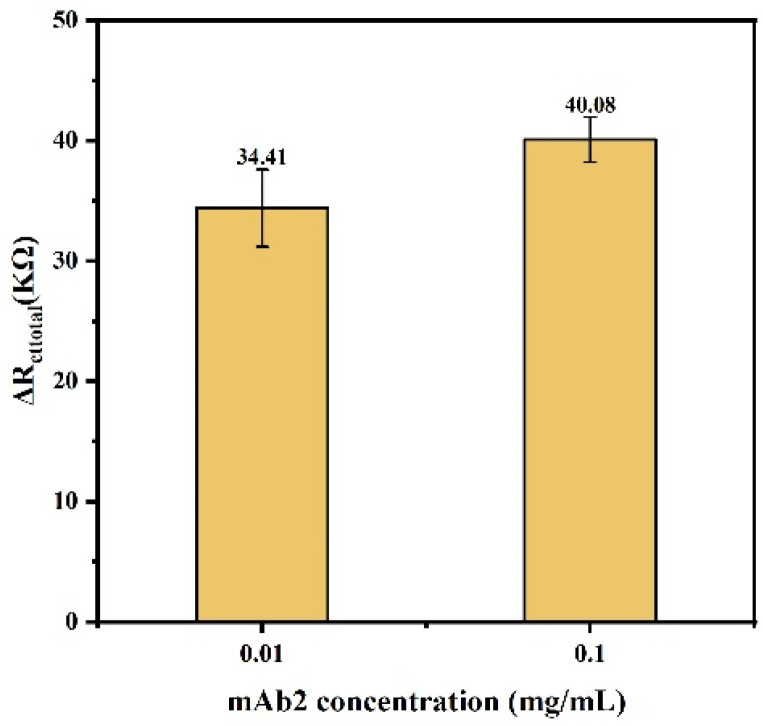
The impedance values after adding different concentrations of mAb2.

In this experiment, 10 μL of 0.1 mg mL^−1^ and 0.01 mg mL^−1^ mAb2 diluted in PBS were selected to bind to tau protein, respectively. During sensor preparation, mAb1 selected the optimized optimal concentration of 0.05 mg·mL^−1^ to capture the target protein as much as possible, and the tau protein concentration was selected as 0.002 mg ml^−1^. The test result is shown in Figure 7.

As can be seen from Figure 7, although the impedance value caused by 0.1 mg mL^−1^ binding to tau protein was larger than that of 0.01 mg mL^−1^, the difference was not significant. Considering the sensor performance and production cost, 0.01 mg·mL^−1^ was selected as the optimal concentration of mAb2.

### 3.4. Detection of Tau Protein

**Figure 8:**
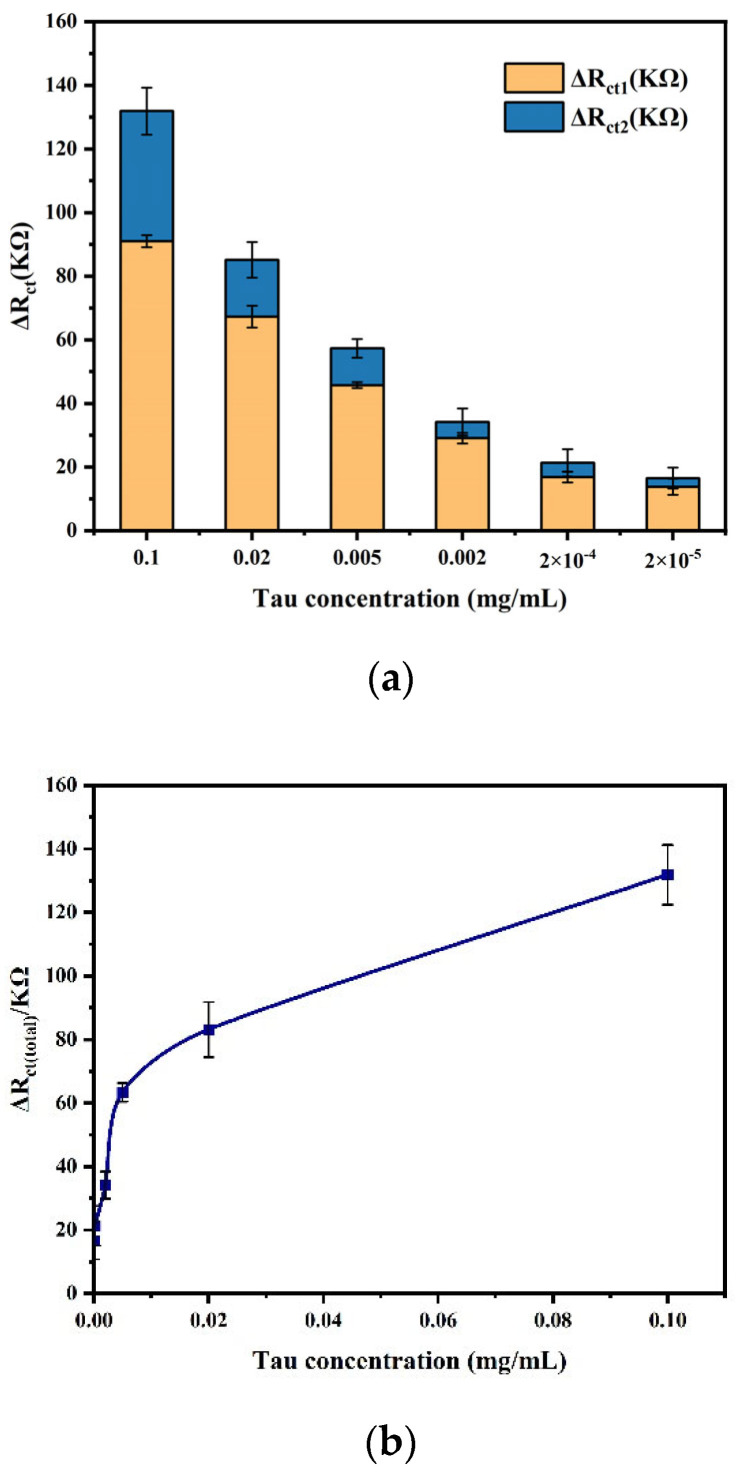
(**a**) Impedance value shift of different concentrations of tau protein. (**b**) Line chart of impedance value shift versus tau protein concentrations.

To evaluate the performance of the fabricated sensor, EIS measurement was performed under optimal experimental conditions for different concentrations of 10 µL tau protein, and the results are shown in Figure 8. ΔR_ct1_ was the difference in impedance before and after the addition of tau protein; ΔR_ct2_ was the difference in impedance before and after the addition of mAb2; and ΔR_cttotal_ was the value of the total change in impedance. The results showed that for each concentration of tau protein, the addition of mAb2 stably amplified the impedance signal. Moreover, ΔR_cttotal_ gradually increased with increasing protein concentration. This was due to the fact that the antibody captured more antigen, which made the structure of the modified layer on the electrode surface more compact, and the subsequent addition of mAb2 further hindered the electron exchange between the Fe(CN_6_)^3−/4−^ probe and the electrode surface. But after reaching a certain concentration, the increasing trend slowed down, which was because the concentration of antibody was fixed and the quantitative antibody captured antigen with its threshold value.

**Figure 9:**
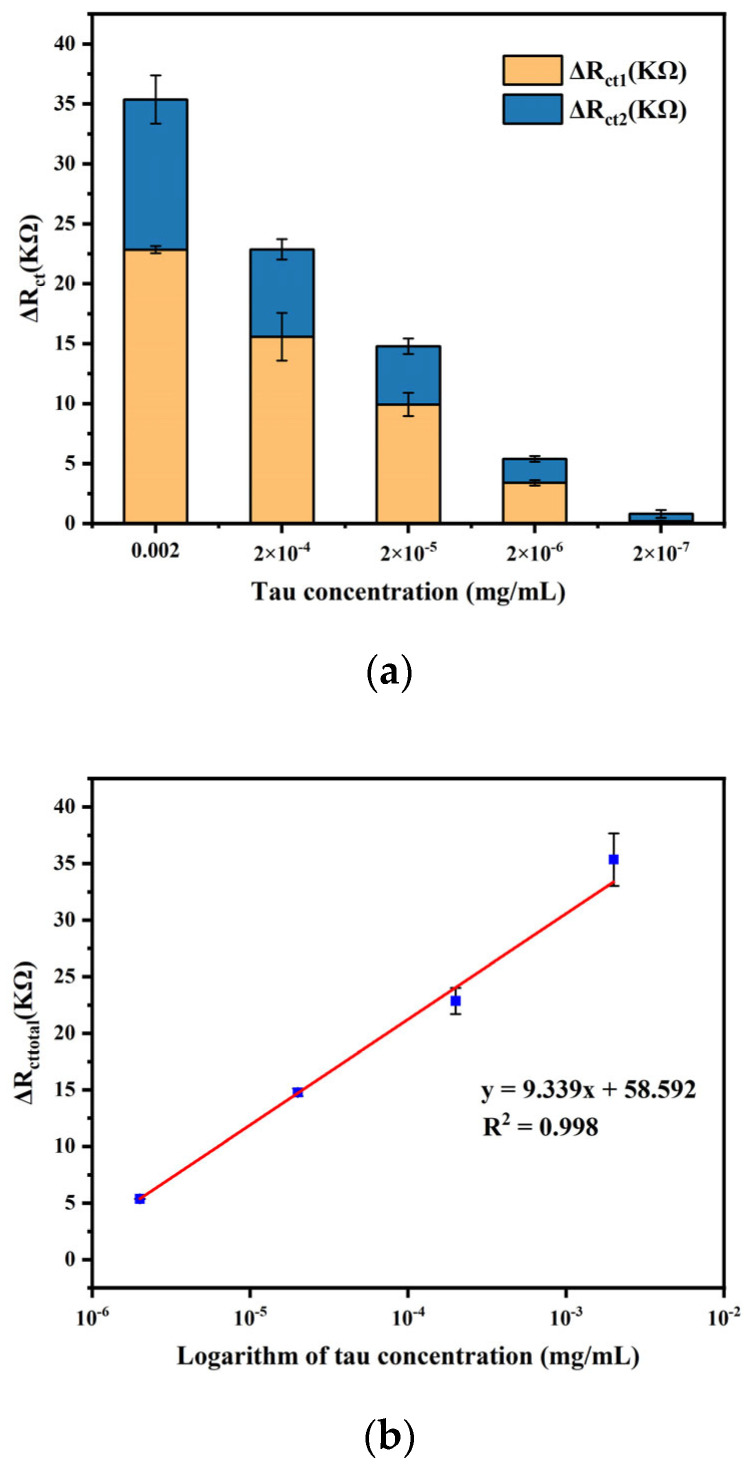
(**a**) Impedance value shift of different concentrations of tau protein. (**b**) Linear relationship of the impedance shift and logarithmic value of tau concentration (Error bar = SD, n = 3 ).

**Figure 10:**
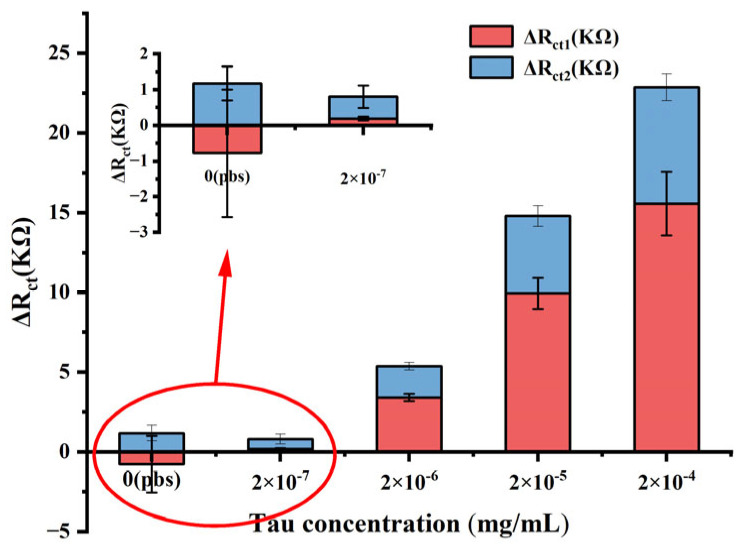
The EIS response of the sensor to tau diluted in PBS.

Due to the extremely low levels of tau protein in the cerebrospinal fluid, the experiment was repeated for lower concentrations, and the results are shown in Figure 9, which are consistent with the results of the previous test. A regression analysis was performed between the total change in impedance and the logarithm of tau concentration. A linear relationship was observed between the total change in impedance and the logarithm of tau concentration, ranging from 2 × 10^−6^ mg mL^−1^ to 2 × 10^−3^ mg mL^−1^, with a regression equation of y = 9.339x + 58.592, and a correlation coefficient of 0.998. In addition, to obtain the limit of detection (LOD), a tau-free solution (PBS in this study) was added to the sensor instead of tau protein, and the detection results are shown in Figure 10. The LOD is calculated using Y_LOD_ = Y_blank_ + 3σ, where Y_blank_ is the response of the blank measurement and σ is the standard deviation of the blank measurement [5,32]. So the LOD of the immunosensor was calculated to 1 × 10^−6^ mg mL^−1^.

### 3.5. Specificity, Stability, and Reproducibility

Specificity. Human serum albumin (HSA), a common standard for plasma protein, is an abundant protein component in blood and cerebrospinal fluid [33]. In this study, HSA was selected to test the specificity of the fabricated biosensor for Tau protein.

**Figure 11:**
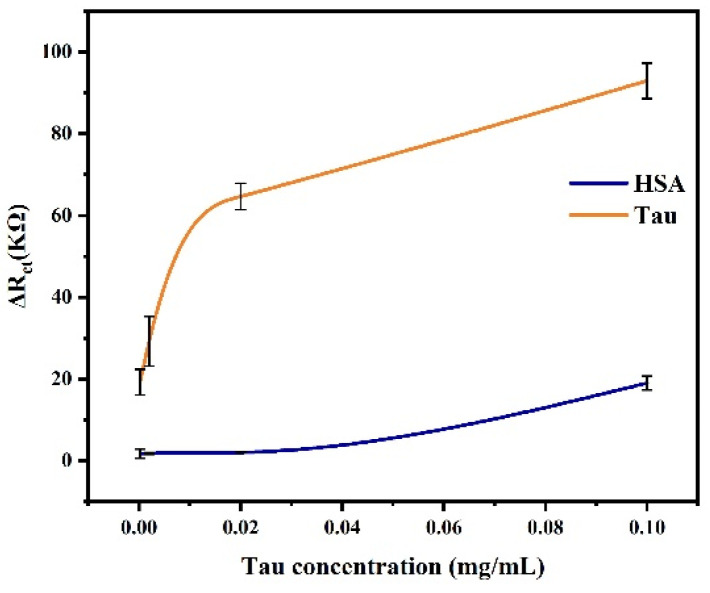
The EIS response of the sensor to tau protein and HSA.

The specificity of the immunosensor was verified through experiments in which the sensor was exposed to HSA and the sensor response was recorded. That is, HSA replaced tau protein as the target protein for capture. As shown in Figure 11, the impedance value of Tau protein was significantly higher than that obtained by HSA. Compared with lower concentrations, the impedance response of HSA at a concentration of 0.1 mg·mL^−1^ increased slightly, which was due to the tension of the HSA solution forming a slightly viscous film on the sensor surface. This value is negligible compared to the impedance response caused by the specific binding of Tau protein to the sensor. The test results showed that the immunosensor has good selectivity and specificity for detecting tau protein.

Stability. The prepared immunosensor was stored for 96 h and then tested for impedance response. Although the impedance value of the sensor was lower than the initial value, the variation was within 15%, which indicates the sensor had satisfied stability over a certain storage time.

**Figure 12:**
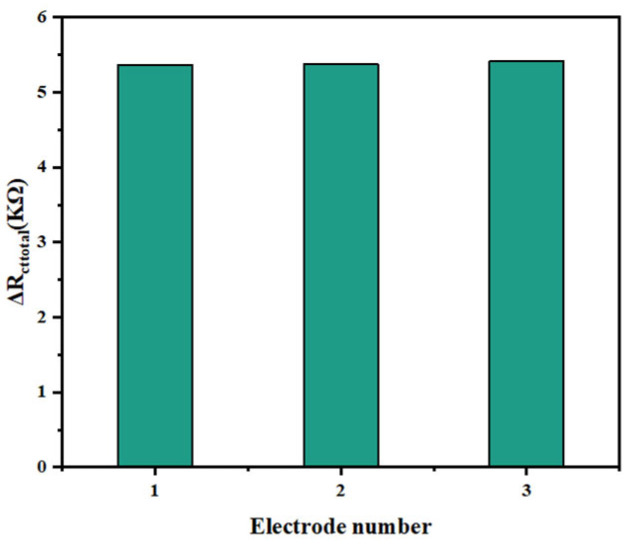
Comparison of impedance values with three different electrodes. The concentration of tau protein was 2 × 10^−6^ mg mL^−1^. (Error bar = SD, n = 3).

Reproducibility. Sandwich immunosensors were fabricated on three different gold electrodes for the detection of the same concentration of the target to evaluate the reproducibility of the biosensor. Figure 12 shows the test results for biosensor reproducibility. As shown in Figure 12, the impedance values of the three biosensors to the target tau protein (2 × 10^−6^ mg mL^−1^) were 5.363 KΩ, 5.378 KΩ, and 5.412 KΩ, with a standard deviation of 2.5%. The experimental data indicated that the fabricated biosensors had good reproducibility.

### 3.6. Sample Analyses

**Figure 13:**
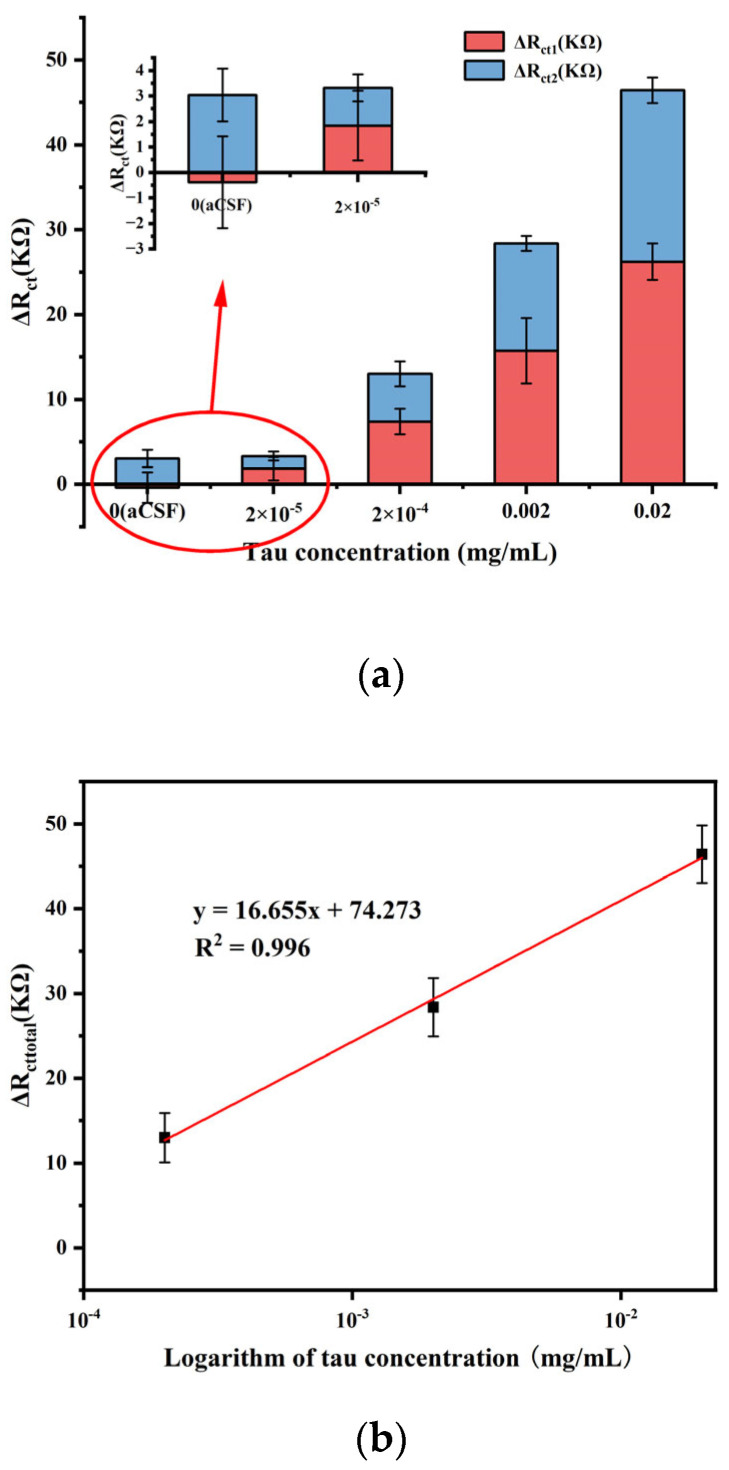
(**a**) The EIS response of the sensor to tau diluted in aCSF. (**b**) Linear relationship of the impedance shift and logarithmic value of tau concentration (Error bar = SD, n = 3).

To evaluate the feasibility of the fabricated immunosensor in practical applications, the sensor was tested on artificial samples. Different concentrations of tau protein diluted with aCSF were analyzed using the sandwich immunosensor, and the results are shown in Figure 12. Among them, ΔR_ct1_ represented the impedance after adding Tau protein, and ΔR_ct2_ represented the resistance after adding mAb2. Comparing Figure 10 and Figure 12, it can be seen that for the same concentration of tau protein (for example, 2 × 10^−4^ mg mL^−1^), the response signal of the artificial sample was smaller than the signal of the standard sample prepared with buffer, which may be due to the complex environment in the sample that will affect the target signal. However, from the data in Figure 12, it can still be seen that the response signal of the sensor increases as the sample concentration increases, indicating that the sensor prepared in this study has the ability to detect samples. As shown in Figure 13, a linear relation was observed between the total change in impedance and logarithm of tau concentration ranging from 1 × 10^−4^ mg mL^−1^ to 0.01 mg mL^−1^, with a regression equation of y = 16.655x + 74.273 and a correlation coefficient of 0.996. And the LOD of the sensor in aCSF was calculated to 1 × 10^−4^ mg mL^−1^, which was higher than the LOD in PBS.

We did a comparative study of the protocol proposed in this study with other tau protein detection methods. As can be seen from Tables S1 and S2 (see Supplementary Materials), the electrochemical immunosensor constructed in this study has huge advantages in terms of cost, detection time, test process, signal acquisition, and sensor preparation. It has been reported that the total tau protein concentration in the cerebrospinal fluid of Alzheimer’s patients is three times the cut-off value of healthy individuals (4.3 pM) [34]. The sensitivity of the electrochemical immunosensor in this study is approximately 18 pM when converted to molar concentration. Therefore, further research is necessary in the future to improve the sensitivity of this immunosensor so that it can distinguish between healthy people and Alzheimer’s patients in future practical applications.

## 4. Conclusions

This study was aimed at developing a simple immune sandwich sensor that could detect tau protein in aCSF. The sensor was firstly modified with mAb1 on the basis of the DTSSP self-assembled monolayer modified onto the surface of the gold electrode, then the tau protein was captured through the specific adsorption between the antigen and the antibody, and finally mAb2 was added to realize the further amplification of the signal. Under the optimal experimental conditions, a linear correlation between the logarithmic value of tau concentrations and charge transfer resistance was found from 2 × 10^−6^ mg mL^−1^ to 2 × 10^−3^ mg mL^−1^ in the sandwich assay. The sensor was fast and sensitive, with a low detection limit of 1 × 10^−6^ mg mL^−1^. Furthermore, the sensor was successfully measuring tau protein in aCSF. This technology could be adapted for the detection of other biomarkers to provide a multiple assay to identify AD progression in a point-of-care setting. This research demonstrated that the sandwich-based electrochemical biosensor has a great potential to be an alternative method for rapid, sensitive, and specific detection of tau protein in clinical samples, and the development of such systems is very important in providing clues towards early and precise diagnosis.

## Data Availability

The raw data supporting the conclusions of this article will be made available by the authors on request.

## Supplementary Materials

The following supporting information can be downloaded at: https://www.mdpi.com/article/10.3390/bios14060279/s1, Table S1: Comparison with ELISA kit; Table S2: Comparison with biosensors in other studies.
