# Supplementary material for: A Simple Sandwich Electrochemical Immunosensor for Rapid Detection of the Alzheimer’s Disease Biomarker Tau Protein
Source: Biosensors (Basel). 2024 May 29;14(6):279. doi: 10.3390/bios14060279 (PMC11202154; doi:10.3390/bios14060279)
Supplement: Supplementary file 1 [file biosensors-14-00279-s001.zip › biosensors-2991282-supplementary.pdf]

Table S1: Comparison with ELISA kit

Methods	Cost	Detection time	Sensitivity	Range	Test procedures	The way to read the signal
Human Tau ELISA Kit, Fluorescent (ab229394)	$725	90min	2.9 pg/ml	5.08 pg/ml - 20800 pg/ml	1. Prepare all reagents, samples, and standards as instructed.<br>2. Add 50 µL standard or sample to appropriate wells.<br>3. Add 50 µL Antibody Cocktail to all wells.<br>4. Incubate at room temperature for 1 hour.<br>5. Aspirate and wash each well three times with 350 µL 1X Wash Buffer<br>6. Add 100 µL of prepared Catch Point HRP Development Solution to each well and incubate for 10 minutes.<br>7. Read fluorescence at Ex/Cutoff/Em 530/570/590 nm.	Measure the optical density (OD) by spectrophotometric method and then calculate the concentration of tau protein in the samples by comparing the OD of the samples to the standard curve.
Mouse MAPτ (Microtubule Associated Protein Tau/Tau Protein) ELISA Kit	$609	3.5h	9.38 pg/ml	15.63 pg/ml - 1000 pg/ml	Standards or samples are added to the micro ELISA plate wells and combined with the specific antibody. Then a biotinylated detection antibody specific for Mouse MAPτ and Avidin-Horseradish Peroxidase (HRP) conjugate are added successively to each micro plate well and incubated. Free components are washed away. The substrate solution is added to each well. Only those wells that contain Mouse MAPτ, biotinylated detection antibody and Avidin-HRP conjugate will appear blue in color. The enzyme-substrate reaction is terminated by the addition of stop solution and the color turns yellow. The optical density (OD) is measured spectrophotometrically at a wavelength of 450 nm ± 2 nm.	

Table S1 (continued)

Methods	Cost	Detection time	Sensitivity	Range	Test procedures	The way to read the signal
Human Phospho Tau (P181) ELISA Kit	$647.4	3h	4.688 pg/ml	7.813 pg/ml - 500 pg/ml	1. Add 0.1 ml of properly diluted sample (Human serum, plasma, tissue homogenates and other biological fluids.) into test sample wells. Seal the plate with a cover and incubate at 37 °C for 90 min.<br>2. Add 0.1 ml of Biotin- detection antibody working solution into the above wells (standard, test sample & zero wells). Add the solution at the bottom of each well without touching the side wall. Seal the plate with a cover and incubate at 37°C for 60 min.<br>3. Add 0.1 ml of SABC working solution into each well, cover the plate and incubate at 37°C for 30 min.<br>4. Add 90 µl of TMB substrate into each well, cover the plate and incubate at 37°C in dark within 10-20 min. (Note: This incubation time is for reference use only, the optimal time should be determined by end user.) And the shades of blue can be seen in the first 3-4 wells (with most concentrated standard solutions), the other wells show no obvious color.<br>5. Add 50 µl of Stop solution into each well and mix thoroughly. The color changes into yellow immediately.<br>6. Read the O.D. absorbance at 450 nm in a microplate reader immediately after adding the stop solution.	Measure the optical density (OD) by spectrophotometric method and then calculate the concentration of tau protein in the samples by comparing the OD of the samples to the standard curve.
High Sensitive ELISA Kit for Tau Protein (MAPT)	$732	3h	6.5 pg/ml	15.6 pg/ml - 1000 pg/ml	1. Prepare all reagents, samples and standards;<br>2. Add 100µL standard or sample to each well. Incubate 1 hours at 37°C;<br>3. Aspirate and add 100µL prepared Detection Reagent A. Incubate 1 hour at 37°C;<br>4. Aspirate and wash 3 times;<br>5. Add 100µL prepared Detection Reagent B. Incubate 30 minutes at 37°C;<br>6. Aspirate and wash 5 times;<br>7. Add 90µL Substrate Solution. Incubate 10-20 minutes at 37°C;<br>8. Add 50µL Stop Solution. Read at 450nm immediately.	
This work	$13.80	2h	1 ng/ml	2 ng/ml - 2000 ng/ml	Incubate diluted tau protein on the developed sensor for 1 hour. Then add mAb2 to the sensor for 1 hour.	Read the impedance value directly

Human Tau ELISA Kit, Fluorescent (ab229394): https://www.abcam.com/products/elisa-kits/human-tau-elisa-kit-fluorescent-ab229394.html
Mouse MAPτ (Microtubule Associated Protein Tau/Tau Protein) ELISA Kit: https://www.elabscience.com/p-mouse_maptau_microtubule_associated_protein_tau_tau_protein_elisa_kit-21020.html
Human Phospho Tau (P181) ELISA Kit: https://www.assaygenie.com/human-phospho-tau-p181-elisa-kit/
High Sensitive ELISA Kit for Tau Protein (MAPT): https://www.cloud-clone.com/products/HEB983Hu.html

Table S2: Comparison with biosensors in other studies

Biosensors	Range	Sensitivity	Detection time	Materials
An enzyme linked aptamer photoelectrochemical biosensor for Tau-381 protein using AuNPs/$MoSe_2$ as sensing material[1]	0.5 fM - 1.0nM	0.3 fM	3.5h	AuNPs/$MoSe_2$ Protein G/AP
An electrochemical immunosensor using gold nanoparticles-PAMAM-nanostructured screen-printed carbon electrodes for tau protein determination in plasma and brain tissues from Alzheimer patients[2]	6 - 5000 pg/ml	1.7 pg/ml	3h 20min	3D-Au-PAMAM EDC-Sulfo-NHS HRP-Dab
Novel biomimetic Prussian blue nanocubes-based biosensor for Tau-441 protein detection[3]	1.09 nM - 2.18nM	0.01 pM	3h	3-Aminophenol Oxalic acid
A tyrosinase-induced fluorescence immunoassay for detection of tau protein using dopamine-functionalized $CuInS_2$/ZnS quantum dots[4]	10 pM - 200nM	9.3 pM	4.5h	$CuInS_2$/ZnS QDs Tyrosinase
This work	2 ng/ml - 2000 ng/ml	18pM	2h 10min	-

As can be seen from Table S2, the sensors reported in the literature all use complex materials such as nanoparticles to achieve lower detection limits. However, the biosensor fabricated in this work achieved a low detection limit of 18pM using only a simple structure. More importantly, the detection time of the sensor only takes about 2 hours, which makes the detection of tau protein fast and simple.

The cut-off value of Tau in cerebrospinal fluid (CSF) of healthy individuals has been reported to be 4.3 pM, while the total tau protein concentration in the CSF of Alzheimer's disease patients is three times the cut-off value of healthy individuals. This significant quantitative difference aids in the diagnosis of disease[5]. Therefore, further research is necessary in the future to improve the sensitivity of this immunosensor based on its simple structure and rapid detection, so that it can distinguish between healthy people and Alzheimer's disease patients in future practical applications.

1. Hun, X.; Kong, X., An enzyme linked aptamer photoelectrochemical biosensor for Tau-381 protein using AuNPs/MoSe2 as sensing material. *Journal of Pharmaceutical and Biomedical Analysis* **2021,** *192*, 113666.
2. Razzino, C. A.; Serafín, V.; Gamella, M.; Pedrero, M.; Montero-Calle, A.; Barderas, R.; Calero, M.; Lobo, A. O.; Yáñez-Sedeño, P.; Campuzano, S.; Pingarrón, J. M., An electrochemical immunosensor using gold nanoparticles-PAMAM-nanostructured screen-printed carbon electrodes for tau protein determination in plasma and brain tissues from Alzheimer patients. *Biosensors and Bioelectronics* **2020,** *163*, 112238.

3. Ben Hassine, A.; Raouafi, N.; Moreira, F. T. C., Novel biomimetic Prussian blue nanocubes-based biosensor for Tau-441 protein detection. *Journal of Pharmaceutical and Biomedical Analysis* **2023,** *226*, 115251.
4. Chen, L.; Lin, J.; Yi, J.; Weng, Q.; Zhou, Y.; Han, Z.; Li, C.; Chen, J.; Zhang, Q., A tyrosinase-induced fluorescence immunoassay for detection of tau protein using dopamine-functionalized CuInS₂/ZnS quantum dots. *Analytical and Bioanalytical Chemistry* **2019,** *411* (20), 5277-5285.
5. Mirzaie, A.; Nasrollahpour, H.; Khalilzadeh, B.; Jamali, A. A.; Spiteri, R. J.; Yousefi, H.; Isildak, I.; Rahbarghazi, R., Cerebrospinal fluid: A specific biofluid for the biosensing of Alzheimer's diseases biomarkers. *TrAC Trends in Analytical Chemistry* **2023,** *166*, 117174.
